# Development of LT-HSC-Reconstituted Non-Irradiated NBSGW Mice for the Study of Human Hematopoiesis *In Vivo*

**DOI:** 10.3389/fimmu.2021.642198

**Published:** 2021-03-25

**Authors:** George Adigbli, Peng Hua, Masateru Uchiyama, Irene Roberts, Joanna Hester, Suzanne M. Watt, Fadi Issa

**Affiliations:** ^1^ Transplantation Research and Immunology Group, John Radcliffe Hospital, Nuffield Department of Surgical Sciences, University of Oxford, Oxford, United Kingdom; ^2^ MRC Molecular Haematology Unit, Weatherall Institute of Molecular Medicine, Radcliffe Department of Medicine, John Radcliffe Hospital, Oxford, United Kingdom; ^3^ Nuffield Division of Clinical Laboratory Medicine, Radcliffe Department of Medicine, John Radcliffe Hospital, University of Oxford, Oxford, United Kingdom; ^4^ Department of Paediatrics, Children’s Hospital, John Radcliffe Hospital, University of Oxford, Oxford, United Kingdom; ^5^ Adelaide Medical School, Faculty of Health and Medical Sciences, University of Adelaide, and Precision Medicine Theme, South Australian Health and Medical Research Institute, Adelaide, SA, Australia

**Keywords:** human immune system mice, human hematological reconstitution *in vivo*, immune reconstitution, red blood cell reconstitution, non-irradiation, genome editing

## Abstract

Humanized immune system (HIS) mouse models are useful tools for the *in vivo* investigation of human hematopoiesis. However, the majority of HIS models currently in use are biased towards lymphocyte development and fail to support long-term multilineage leucocytes and erythrocytes. Those that achieve successful multilineage reconstitution often require preconditioning steps which are expensive, cause animal morbidity, are technically demanding, and poorly reproducible. In this study, we address this challenge by using HSPC-NBSGW mice, in which NOD,B6.SCID IL-2r*γ* ^-/-^Kit^W41/W41^ (NBSGW) mice are engrafted with human CD133^+^ hematopoietic stem and progenitor cells (HSPCs) without the need for preconditioning by sublethal irradiation. These HSPCs are enriched in long-term hematopoietic stem cells (LT-HSCs), while NBSGW mice are permissive to human hematopoietic stem cell (HSC) engraftment, thus reducing the cell number required for successful HIS development. B cells reconstitute with the greatest efficiency, including mature B cells capable of class-switching following allogeneic stimulation and, within lymphoid organs and peripheral blood, T cells at a spectrum of stages of maturation. In the thymus, human thymocytes are identified at all major stages of development. Phenotypically distinct subsets of myeloid cells, including dendritic cells and mature monocytes, engraft to a variable degree in the bone marrow and spleen, and circulate in peripheral blood. Finally, we observe human erythrocytes which persist in the periphery at high levels following macrophage clearance. The HSPC-NBSGW model therefore provides a useful platform for the study of human hematological and immunological processes and pathologies.

## Introduction

The ability to conduct experiments on human cells and tissues in small animal models holds great value for immunological research. Humanized immune system (HIS) mice are produced by successful transplantation and engraftment of human hematopoietic cells into immunodeficient mice. These *in vivo* models provide a powerful tool for the study of long-term human hematopoiesis and for the preclinical investigation of transplant rejection, cancer, autoimmunity and infection.

Immunodeficient strains such as the non-obese diabetic (NOD)/severe combined immunodeficiency (SCID)/interleukin-(IL)2 receptor gamma chain null (IL2r*γ* ^-^/^-^) mouse (NSG) (1) have profound deficiencies of innate and adaptive immunity and impaired capacity to repopulate the murine bone marrow, both of which enhance engraftment of human hematopoietic stem cells (HSCs) (2). Despite this, attainment of high levels of human leucocyte chimaerism requires preconditioning and adoptive transfer of large numbers of HSCs (3, 4). To address this, the NOD,B6.SCID IL-2r*γ* ^-^/^-^Kit^W41/W41^ (NBSGW) immunodeficient mouse strain was developed by adding *Kit^W41/W41^* alleles to the NSG (5). This allele affords a competitive advantage to transplanted HSCs through a loss-of-function mutation of the tyrosine kinase motif of the stem cell factor receptor, c-Kit. A recent study demonstrated successful humanization of NBSGW mice using HSCs sourced from human umbilical cord blood (hUCB), bone marrow and mobilized peripheral blood (6). The majority of HSC-based HIS models in current use utilize human umbilical cord blood (hUCB)-derived cells, with HSC isolation based on expression of CD34. However, because HSC yields from individual hUCB units are both highly variable and expensive to procure and isolate (7), there is a need for mouse models that can support immune reconstitution following transplantation of low numbers of primitive HSCs. In NBSGW mice, successful humanization with high levels of chimaerism is achievable following transplantation of 0.25x10^6^–1x10^6^ CD34^+^ HSCs (8). In contrast to CD34, CD133, a pentaspan transmembrane protein on human HSCs, is a key biomarker, expressed on the surface of human HSCs before and then with CD34 (9), and used in place of CD34 for the isolation and characterization of human HSCs (10, 11). In UCB, the CD133^+^ subpopulation has been shown to be particularly enriched for long-term (LT)-HSCs and the CD133^neg^ fraction to lack LT-HSCs (12, 13). Additionally, the CD133^+^ population includes a small fraction of CD34^neg^ LT-HSCs which have the capacity for self-renewal (13), are increasingly appreciated as the determinants of successful transplantation (14), and are likely discarded where transplantation is based only on CD34 selection. In NOG mice, isolation based on CD133 positivity has been shown to improve engraftment of UCB HSCs compared with CD133^-^ HSCs (15).

For immunological research, combined reconstitution of functional human adaptive and innate immune cells is desirable to enable more accurate experimental representation of human immunity to be achieved *in vivo*. Currently available models demonstrate cellular tropism, with biased reconstitution of particular populations of cells. Peripheral blood leucocyte (PBL) HIS models are commonly used examples, which are typically T cell-biased (16). HSC-based HIS models tend to support myeloid cell engraftment but are typically skewed toward reconstitution of immature B cells (17) which fail to mature into functional mature B cells.

T cells that develop within the thymi of HIS mice include CD4^+^ and CD8^+^ cells with broad Vβ distribution together with regulatory T cells (Tregs) and *γ*δ T cells (18). To date, successful intrathymic *de novo* T cell development following human HSC transplantation has required the use of irradiated newborn mice (19–21). When this has also been demonstrated in adult mice, its achievement requires irradiation and weekly injections of human Fc-IL7 fusion protein, which conferred the additional effect of diminishing the human B cell population (3).

Human erythropoiesis is not well supported in humanized mouse models. Within the NBSGW mouse, bone marrow-based erythropoiesis occurs with complete maturation, enucleation and globin gene expression (8). However, survival of mature human erythrocytes in the peripheral blood does not occur, likely as a result of murine macrophage-mediated erythrocyte phagocytosis.

In this study we harness stemness properties of CD133^+^ hUCB LT-HSCs to achieve successful irradiation-independent human hematopoietic reconstitution in NBSGW mice using low doses of HSCs. The model is technically easy to use and achieves robust multilineage reconstitution of lymphoid and myeloid human cells which persist long-term. For decades, achieving this has challenged several of the available HIS mouse models, which are unable to support both engraftment of all lymphocytes and myeloid cells and maturation and survival into the long-term (22). Human thymocytes develop in a humanized thymic microenvironment and both naïve and memory CD4^+^ and CD8^+^ T cells repopulate in the periphery. Both immature and mature B cells are present, which are antibody class-switching and functional. Finally, we identify human erythropoiesis within the bone marrow.

## Materials and Methods

### Cell Isolation

hUCB was collected from the John Radcliffe Hospital, Oxford, UK or provided *via* the NHS Cord Blood Bank, London and used with informed, written pre-consent and ethical approval from the South Central Oxford C and Berkshire Ethical Committees (# 15/SC/0027) and the Oxfordshire Research Ethics Committee B (#07/H0605/130), in accordance with the Helsinki Declaration of 1975, as revised in 2008.

Mononuclear cells (MNCs; density <1.077g/ml) were isolated by density gradient centrifugation no more than 24 hours after hUCB collection. Human CD133^+^/hCD34^+^ hematopoietic stem and progenitor cells (HSPCs) were enriched by magnetic bead selection using the human CD133/hCD34 direct microbead kits (MACS, Miltenyi Biotec GmbH) and cryopreserved until use (23, 24). Purity was routinely assessed by flow cytometry and only cell isolates with >90% hCD133^+^ or hCD34^+^ cell purity were used for experiments. PBMCs were isolated from leucocyte cones obtained from healthy donors (NHS Blood and Transplant [NHSBT] UK) by LSM1077 (PAA) gradient centrifugation.

### Cell Dose, Preparation and Injection Into Mice

NOD,B6.SCID Il2r*γ* ^-/-^ Kit^W41/W41^ (NBSGW) mice were obtained from the Jackson Laboratory and then bred and housed in the Biomedical Services Unit of the John Radcliffe Hospital (Oxford, UK) in individually ventilated cages. All experiments were performed using protocols approved by the Animal Care and Ethical Review Committee at the University of Oxford, in accordance with the UK Animals (Scientific Procedures) Act 1986 and under the PPL P8869535A. Cells were quantified for injection using Countbright absolute counting beads (Molecular Probes) by flow cytometry (23). HSPCs were injected intravenously at doses of between 1,000-250,000 CD133^+^ cells, suspended in 200μl of IMDM/1%HSA (human serum albumin) into non-irradiated adult (5-16 weeks of age) recipient male or female mice. Mice were regularly bled for human leucocyte reconstitution assessment. 20-22 weeks after HSPC injection, peripheral blood, spleen, thymus and bone marrow were harvested. Engraftment was defined as >0.1% hCD45^+^ cell chimaerism in bone marrow at final harvest (25). A separate group of 8-10 week old male or female mice were injected intravenously with 5x10^6^ human PBMCs suspended in 200μl of RPMI 1650. 4-6 weeks after PBMC injection peripheral blood, spleen and thymus were harvested.

### Sample Processing

Blood, spleen, both femurs and thymus were harvested. Spleen and thymus were mashed and filtered using a 70µm nylon filter, washed with MACS buffer, and red blood cells lysed (BD Pharmlyse), followed by repeated filtering. Femurs were cleaned, crushed with the back end of a 10ml syringe plunger and filtered through a 70µm nylon filter with MACS buffer to wash out bone marrow cells. 1/50^th^ of a femur was taken for RBC staining and remaining bone marrow was lysed with RBC lysis buffer (BD Pharmlyse). Cells were then washed and filtered again. 10µl of peripheral blood was taken for RBC staining (no RBC lysis) and 50µl of blood was taken for each of the remaining stains after undergoing RBC lysis with BD Pharmlyse buffer, followed by washing with MACS buffer.

### Flow Cytometric Analysis

Bone marrow, spleen, thymus and blood single cell suspensions prepared as described above were used for flow cytometry. The antibodies against human and mouse cell surface antigens are detailed in **Supplementary Table 1**. Briefly, cells were incubated with a mixture of fluorescently labeled antibodies diluted in FACS buffer for 30 minutes on ice. Cells were washed once, resuspended in FACS buffer and acquired immediately on a BD FACSCanto flow cytometer (BD Biosciences). 7-AAD (eBioscience) was added to the antibody mix to distinguish live and dead cells. Data were analyzed using FACSDiva (BD) and FlowJo software (TreeStar Inc.).

### Human Skin Transplantation

Human tissue samples were obtained with full informed written consent and with ethical approval from the Oxfordshire Research Ethics Committee (REC B), under study number 07/H0605/130. In 2 separate experiments, 14 non-irradiated adult NBSGW mice were injected with CD133^+^ hUCB cells suspended in 200μl of IMDM/1%HSA and 11-13 weeks after humanization, received a 1cm x 1cm allogeneic split-thickness human skin graft harvested from excess abdominal tissue used for reconstructive surgery (as previously described (26)). 6 additional mice, which did not receive skin transplants were observed to 100 days to compare incidence of adverse effects. At the time of skin transplantation, multilineage leucocyte reconstitution can be observed in the peripheral blood (data not shown). Grafts were monitored for clinical features of rejection and mice were sacrificed when these were observed. In the absence of rejection grafts were harvested 100 days after transplantation. Spleens were harvested concurrently and splenocytes, processed (as above) and analyzed by FACS for the frequency of class-switched memory B cells. Additionally, blood was harvested and serum separated from cells following centrifugation at 1300rpm for 10 minutes. IgD and IgG expression was assessed by cytometric bead array (Biolegend Legendplex Human Ig Isotyping Panel (8-plex)) according to the manufacturer’s instructions. Data were analyzed using FACSDiva (BD) and FlowJo software (TreeStar Inc.).

### Phagocyte Depletion

For the phagocyte depletion assays, mice were injected with 50,000 CD34^+^ hUCB HSPCs. On week 10, mice were checked for human leucocyte reconstitution and allocated to the clodronate and PBS-treated groups to provide equal distribution of human leucocyte reconstitution levels. Mice received either 200μl clodronate liposomes (at 5mg clodronate per ml; Liposoma B.V.) or 200μl PBS intraperitoneally. Five (5) days after injection, mice were analyzed for mouse macrophage depletion and human RBC levels in the peripheral blood (27). The bone marrow was then harvested at day 6 post-clodronate injection.

### Data Analysis and Statistics

Statistical analyses were performed using GraphPad Prism 5 software using the Mann Whitney test and paired t-test as described in each figure legend. A p value of <0.05 was considered statistically significant. Median values are shown, unless stated otherwise.

## Results

### Human Leucocyte Reconstitution and HSPC Engraftment in HSPC-NBSGW Mice

**Figure 1:**
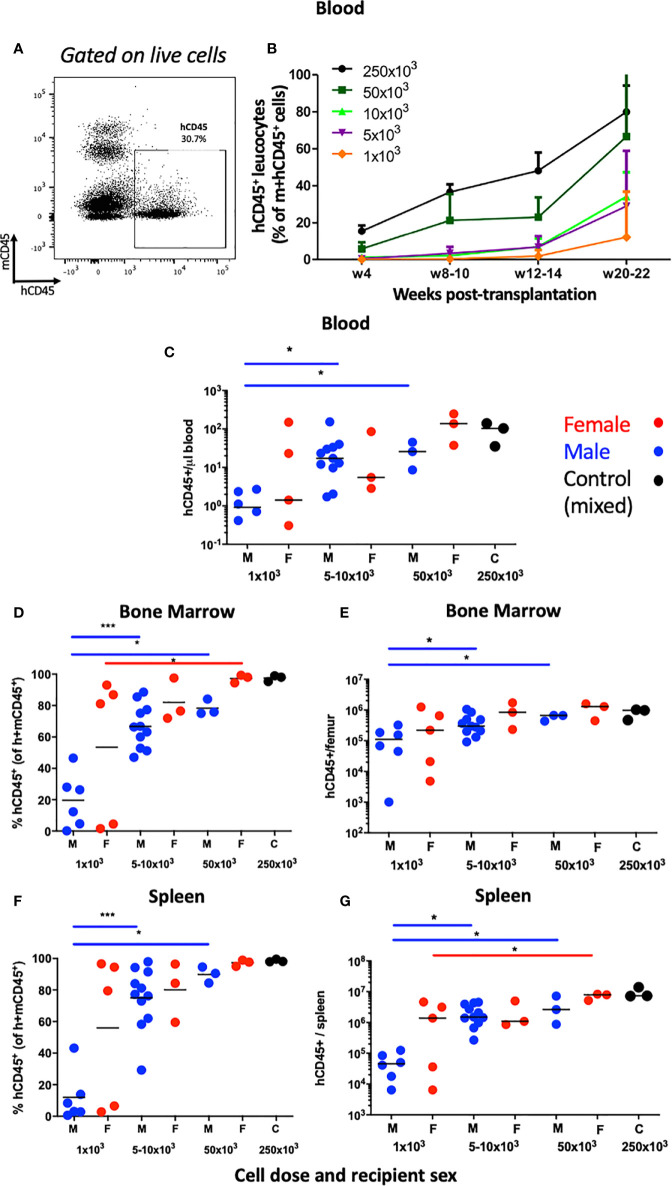
Human leucocyte reconstitution in HSPC-NBSGW mice. **(A)** Representative flow cytometric analysis of mCD45^+^ vs hCD45^+^ cells in the peripheral blood of non-irradiated NBSGW recipients 20-22 weeks after i.v. injection with 1x10^3^ hCD133^+^ hUCB HSPCs. **(B)** Frequencies of hCD45^+^ leucocytes in the blood at indicated time points according to dose of injected HSPCs (mean and SD shown). **(C)** Absolute numbers of human CD45^+^ leucocytes per microliter of blood in male and female recipient mice, based on number of injected HSPCs. **(D–G)** Human CD45^+^ leucocyte frequencies (percentage of total mouse plus human CD45^+^ cells) and corresponding absolute numbers per, bone marrow of one femur and spleen in male and female recipient mice, based on number of injected HSPCs. M- male (blue symbols), F- female (red symbols), 250x10^3^ C – control group (mixed sex). Statistical significance was assessed using a Mann Whitney test (*p<0.05, ***p<0.001). In **(C)**, two points fall below the cut-off shown for 1x10^3^ cells infused. Non-statistical bars represent median values.

**Figure 2:**
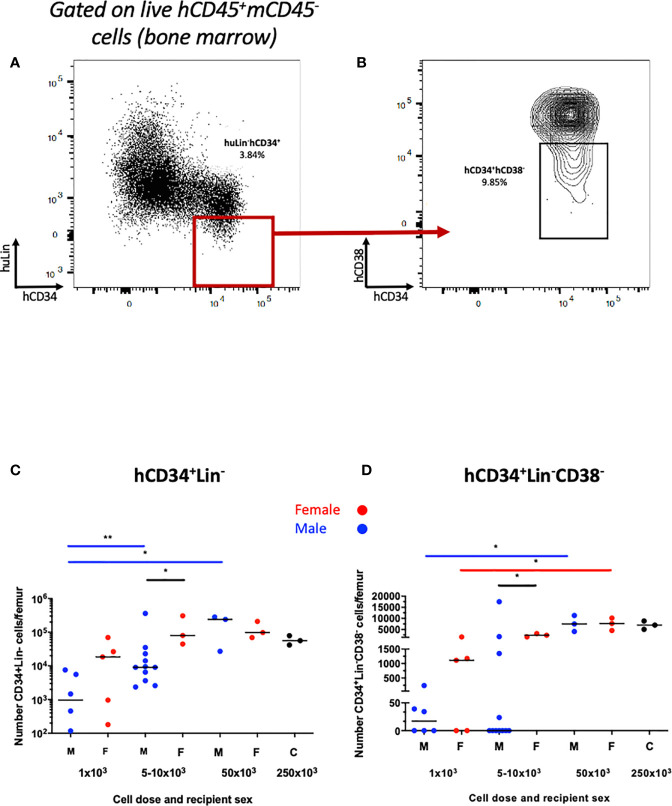
Human HSPC engraftment in the bone marrow of HSPC-NBSGW mice. Representative dot plots of **(A)** hCD34^+^Lin^-^ and **(B)** hCD34^+^Lin^-^hCD38^low\-^ HSPCs in the bone marrow. Number of **(C)** hCD34^+^Lin^-^ and **(D)** hCD34^+^Lin^-^hCD38^low\-^ cells per femur in male and female recipients at the point of harvest (20-22 weeks). M- male (blue symbols), F- female (red symbols), 250x10^3^ – control group (mixed sex). Statistical significance was assessed using a Mann Whitney test (*p<0.05, **p<0.01). Median values shown.

We first examined human hematopoietic chimaerism in the peripheral blood following transplantation of hUCB CD133^+^ HSCs (≥1x10^3^ CD133^+^ cells injected) into non-irradiated NBSGW mice (referred to herein as HSPC-NBSGW mice). Following isolation, >96% of CD133^+^ cells were strongly positive for CD34, and included a small number of the rare but highly potent CD133^+^CD34^-^ LT-HSCs (**Supplementary Figure 1a**). Successful humanization was demonstrated by a dose-dependent increase in human CD45^+^ cell chimaerism in the bone marrow, spleen and peripheral blood by 20-22 weeks (**Figures 1A, B**; **Supplementary Tables 2** and **3**, **Supplementary Figure 1B**). Engraftment was greatest in the bone marrow (mean ± SD from 35.0±36.2 to 97.4±1.9%) and spleen (from 32.0±39.4 to 98.5±0.9%) (**Supplementary Table 2**). Robust engraftment was also present in the peripheral blood (from 12.1±24.7 to 80.0±14.2%), with higher chimaerism than described in comparable irradiated HSPC mice similarly humanized intravenously with hUCB HSCs (28). At all doses administered, reconstitution in the blood demonstrated no signs of declining at 20-22 weeks (**Figure 1B**). Recipient sex did not significantly affect chimaerism in the bone marrow, spleen or peripheral blood (**Figures 1C–G**, **Supplementary Figure 1C**), however we identified a trend consistent with other immunodeficient strains including NSG mice in which lower levels of human CD45^+^ cell engraftment are seen in the bone marrow of male recipients. Engraftment of a sizeable proportion of Lin^-^CD34^+^ and Lin^-^CD34^+^CD38^lo/-^ cells in the bone marrow was seen and suggests a pool of stable, self-renewing HSPCs is maintained for at least 20 weeks (**Figures 2A–D**). Relative engraftment of these cells was greater in the bone marrow of female as compared to male recipients when 5-10x10^3^ HSPCs were transplanted (**Figures 2C, D**). We previously demonstrated functional self-renewal through engraftment of human leukocytes in NBSGW mice secondarily transplanted with bone marrow cells from HSPC-NBSGW mice (29),

### B Cells Dominate Multilineage Human Leucocyte Engraftment

**Figure 3:**
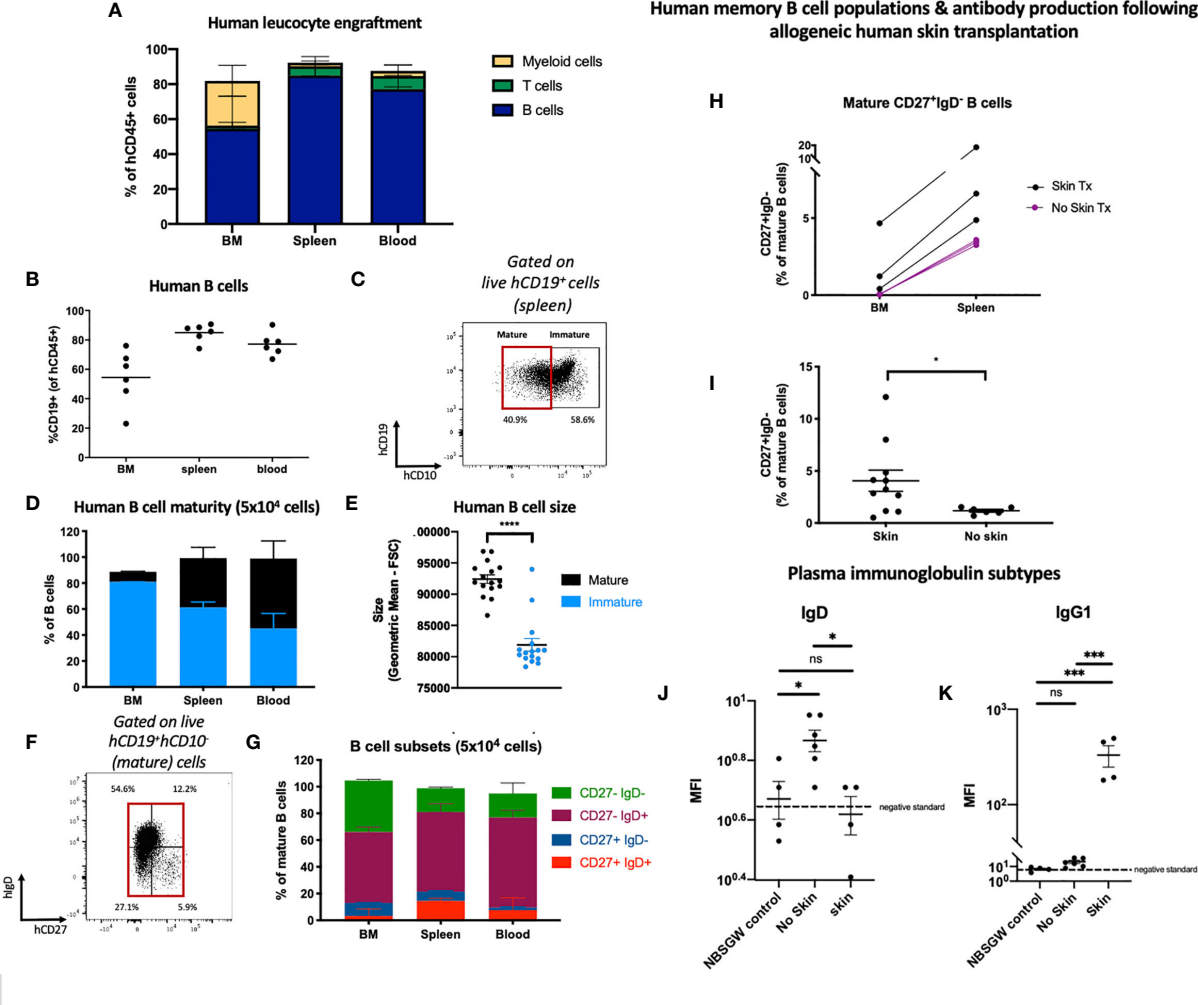
Human B cell engraftment and development in HSPC-NBSGW mice. **(A)** Frequencies of human B cells, myeloid cells and T cells in the bone marrow, spleen and blood of HSPC-NBSGW mice 20-22 weeks after cell injection (50x10^3^ cells). **(B)** Frequency of hCD19^+^ cells in the bone marrow (BM), spleen and peripheral blood (50x10^3^ cells). Bars indicate median values. **(C)** Representative flow cytometric analysis of CD10 expression on human CD45^+^CD19^+^ B cells in spleens harvested at 20-22 weeks. **(D)** Frequencies of mature (CD10-) and immature (CD10+) human B cells in the bone marrow, spleen and blood. **(E)** Sizes of mature vs immature human splenic B cells (determined by forward scatter (FSC). **(F)** Representative flow cytometric analysis and **(G)** corresponding frequencies of mature (CD10^-^) B cell subsets in the bone marrow, spleen and blood, based on IgD and CD27 expression. The red box indicates mature cells [as gated in **(C)**]. **(H)** Frequencies of IgD-negative memory B cells in the femurs and spleens of HSPC-NBSGW mice which received allogeneic split-thickness human skin grafts 11 weeks after humanization and were sacrificed at the time of skin rejection (skin Tx) or 100 days after transplantation (no skin Tx). **(I)** Frequencies of class-switched memory B cells in the spleens of mice which did (skin) and did not (no skin) receive allogeneic split-thickness human skin grafts. **(J, K)** Geometric mean fluorescence intensity (MFI) of plasma **(J)** human IgD and **(K)** human IgG1 in NBSGW (control), HSPC-NBSGW (no skin) and human skin-transplanted HSPC-NBSGW (skin) mice. Dashed lines represent negative standards (background fluorescence). Bars represent median + IQR **(A, D, G)**, mean + SEM **(E, I)** and median **(H)** values. Statistical significance was assessed using the paired t test **(D)**, two-tailed Mann-Whitney test **(I)**, and ordinary one-way ANOVA with Tukey’s multiple comparisons test **(J, K)** (*p<0.05; ***p<0.001; ****p<0.0001; ns p>0.05).

We observed multilineage human leucocyte engraftment in the bone marrow, spleen and peripheral blood (**Figure 3**; **Supplementary Figure 2**). B cells engrafted with the greatest frequency (**Figures 3A, B**; **Supplementary Figures 2A–C**, followed by myeloid and T cells (**Supplementary Tables 2** and **3**). In mice receiving 1x10^3^ hUCB HSPCs, hCD19^+^ B cell engraftment was higher in females (**Supplementary Figure 2d**). No other sex-specific differences in leucocyte subset engraftment were observed (**Supplementary Figures 2D–F**). We also identified human natural killer cells, which were most populous in the peripheral blood. (**Supplementary Figures 2G, H**)

B cell hematopoiesis was assessed by examining the developmental stages of B cells in the bone marrow, spleen and peripheral blood 20-22 weeks after humanization (**Figure 3B**). Within the bone marrow, immature B cells expressing CD10 were most populous (30) (**Figures 3C, D**), contrasting with increasing frequencies of larger CD10^-^ mature B cells in the spleen and blood (**Figures 3D, E**), supporting early observations of the memory phenotype being associated with greater-sized B cells (31). This correlates with human bone marrow, which contains more CD10^+^ cells (with higher CD10 density) than both hUCB CD34^+^ cells and G-CSF-mobilized peripheral blood (30). Next, we analyzed mature B cell phenotypes. As expected, only a small proportion of CD10^-^ B cells populated the bone marrow and the majority were naïve, lacking CD27 expression (**Figures 3F–G**). Of these, almost half were CD27^-^IgD^-^ double-negative B cells (**Figure 3G**), a heterogenous population described as early-stage bone marrow cells (32), and more recently as memory precursor, extracellular antibody secreting cell precursor, and atypical/tissue based memory cells, with activated phenotypes in inflammatory diseases (33). As in humans, antigen-inexperienced CD27^-^IgD^+^ B cells formed the other major mature B cell population in the bone marrow (34) (**Figure 3G**). Within the spleen a greater proportion of mature B cells was observed and among them both unswitched (CD27^+^IgD^+^) and class-switched memory B cells (CD27^+^IgD^-^) (**Figures 3F–G**). This reflected the notion that class switch recombination (CSR) occurs predominantly (although not exclusively) in germinal center (secondary lymphoid organ) B cells, initiated by activation-induced cytidine deaminase (35). To assess the capacity for isotype switching to be stimulated *in vivo*, we introduced an antigen challenge by transplanting allogeneic human skin onto HSPC-NBSGW mice 11-13 weeks after humanization. At the point of skin allograft rejection (or after 100 days), we analyzed the phenotypes of human leucocytes, splenic B cells and immunoglobulin subtypes in peripheral serum (**Figures 3H–K**; **Supplementary Figures 2i–k**). We found a significantly higher frequency of CD27^+^IgD^-^ memory B cells (**Figure 3I**), together with lower serum IgD and higher IgG1 levels (**Figures 3J, K**) in mice exposed to allogeneic skin transplants compared with mice not exposed. This indicates the potential for specific human B cell responses to be mounted in response to antigen challenge within this model.

Altogether, these findings demonstrate that following transplantation of hUCB CD133^+^ HSPCs a continuous process of human B cell development and functional maturation occurs within the primary and secondary lymphoid organs of HSPC-NBSGW mice.

### Thymic Development of Human T Cells in HSPC-NBSGW Mice

**Figure 4:**
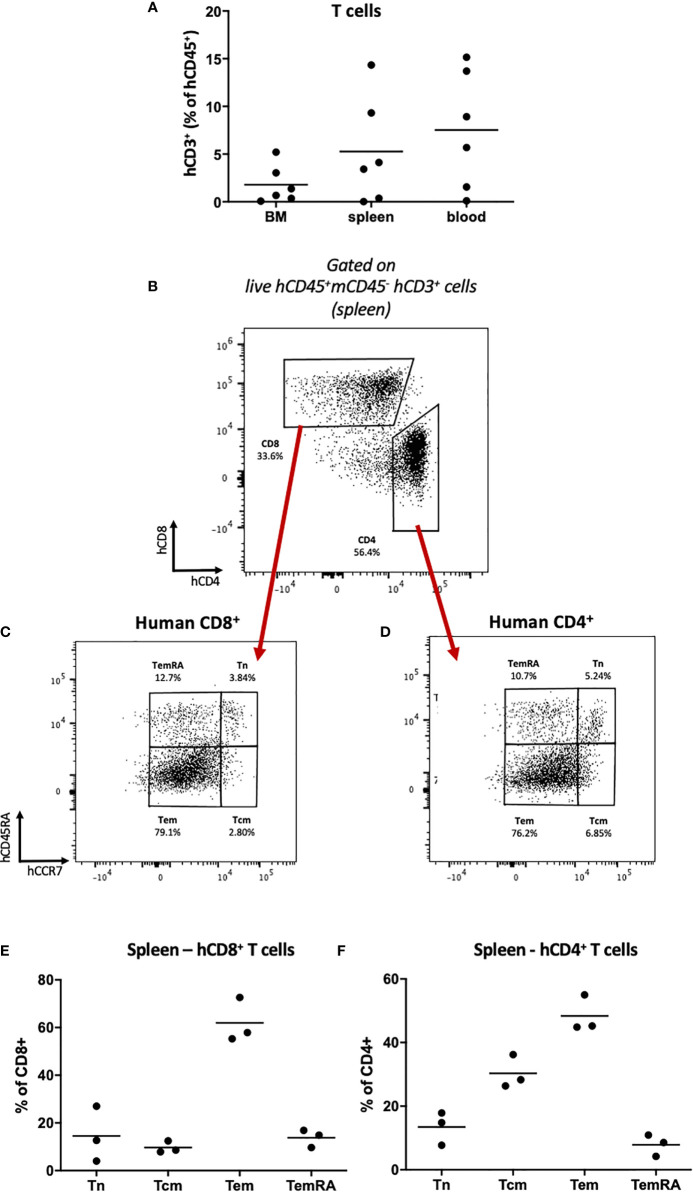
Human T cell repopulation in HSPC-NBSGW mice **(A)** Frequencies of human CD3^+^ T cells in the bone marrow, spleen and blood of HSPC-NBSGW mice 20-22 weeks after cell injection, as determined by flow cytometry (50x10^3^ HSPC dose shown). **(B–D)** Representative flow cytometric analysis of **(B)** CD4 and CD8 expression on live human CD45^+^CD3^+^ splenic T cells, **(C)** CD8^+^ and **(D)** CD4^+^ T cell subsets, determined by surface expression of CD45RA and CCR7. **(E, F)** Frequencies of CD8^+^ **(D)** and CD4^+^ **(E)** splenic T cell subsets: naïve (Tn; hCD45RA^+^hCCR7^+^), Tcm (hCD45RA^-^hCCR7^+^), Tem (hCD45RA^-^hCCR7^-^) and TemRA (hCD45RA^+^hCCR7^-^) (250x10^3^ HSPC dose shown). Bars indicate median values.

Having identified long-term engraftment of T cells (**Figure 3A**; **Supplementary Figure 2**), we sought to investigate whether persistence of these cells is also supported by continuous hematopoiesis and thymic development. We first assessed T cell subset profiles within the bone marrow, spleen and blood. Transplantation of high doses (≥50x10^3^) of HSPCs produced robust engraftment of both CD8^+^ and CD4^+^ T cells, predominantly in the spleen and peripheral blood (**Figures 4A, B**). At these doses we identified the major T cell subtypes within both CD8^+^ and CD4^+^ T cell populations: 1) central memory (Tcm), 2) effector memory (Tem), 3) T effector memory re-expressing CD45RA (TemRA) and 4) naïve (Tn) (**Figures 4C, D**; **Supplementary Table 4**). Tem and Tcm cells were the most populous subtypes, however a significant proportion of CD45RA-expressing cells also persisted long-term (**Figures 4C–F**; **Supplementary Figure 3D**, **Supplementary Table 4**). We found T cell reconstitution in HSPC-NBSGW mice to more accurately reproduce the average human peripheral blood profiles than in NBSGW mice humanized with peripheral blood mononuclear cells (PBMCs), which fail to reconstitute CD45RA-expressing T cells and are instead composed entirely of Tem and Tcm (**Supplementary Figures 3A–D**).

**Figure 5:**
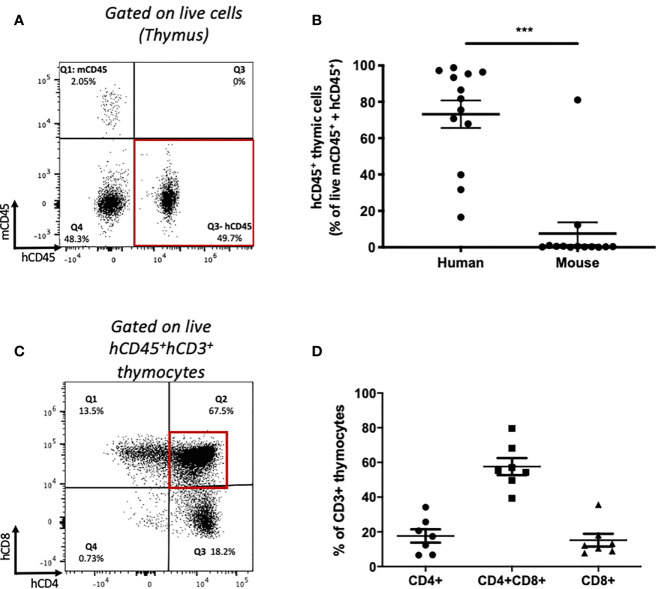
Thymic human leucocyte engraftment and development of human thymocytes in HSPC-NBSGW mice. **(A)** Representative flow cytometric plot and **(B)** corresponding frequencies of human and mouse CD45^+^ cells (as percentage of mCD45 + hCD45 cells) in the thymi of HSPC-NBSGW mice 20-22 weeks after cell injection (50x10^3^ dose shown). **(C)** Representative flow cytometric analysis and **(D)** corresponding frequencies of single-positive CD4^+^, CD8^+^ and double-positive CD4^+^CD8^+^ human thymocytes. Bars represent the mean ± SEM. Statistical significance was assessed using paired t tests (***p<0.001).

To identify whether *de novo* T cell development from transplanted HSPCs occurs in this model, we analyzed human and mouse leucocyte populations in the thymi of recipient mice humanized with HSPCs or PBMCs (**Figure 5**). The majority of thymic cells were human CD45^+^ leucocytes (**Figures 5A, B**) expressing CD3, together with a small population of CD19^+^ cells (**Supplementary Figure 4A**). The majority of CD3^+^ cells were CD4^+^CD8^+^ double-positive (DP) thymocytes (**Figures 5C, D**) with an average frequency equivalent to those seen in thymus biopsies from human infants (36). In contrast, following humanization with mature PBMCs, no double-positive T cells were identified within the thymus (**Supplementary Figures 4B–C**).

### Successful Engraftment and Reconstitution of Phenotypically Distinct Subsets of Innate Myeloid Cells

**Figure 6:**
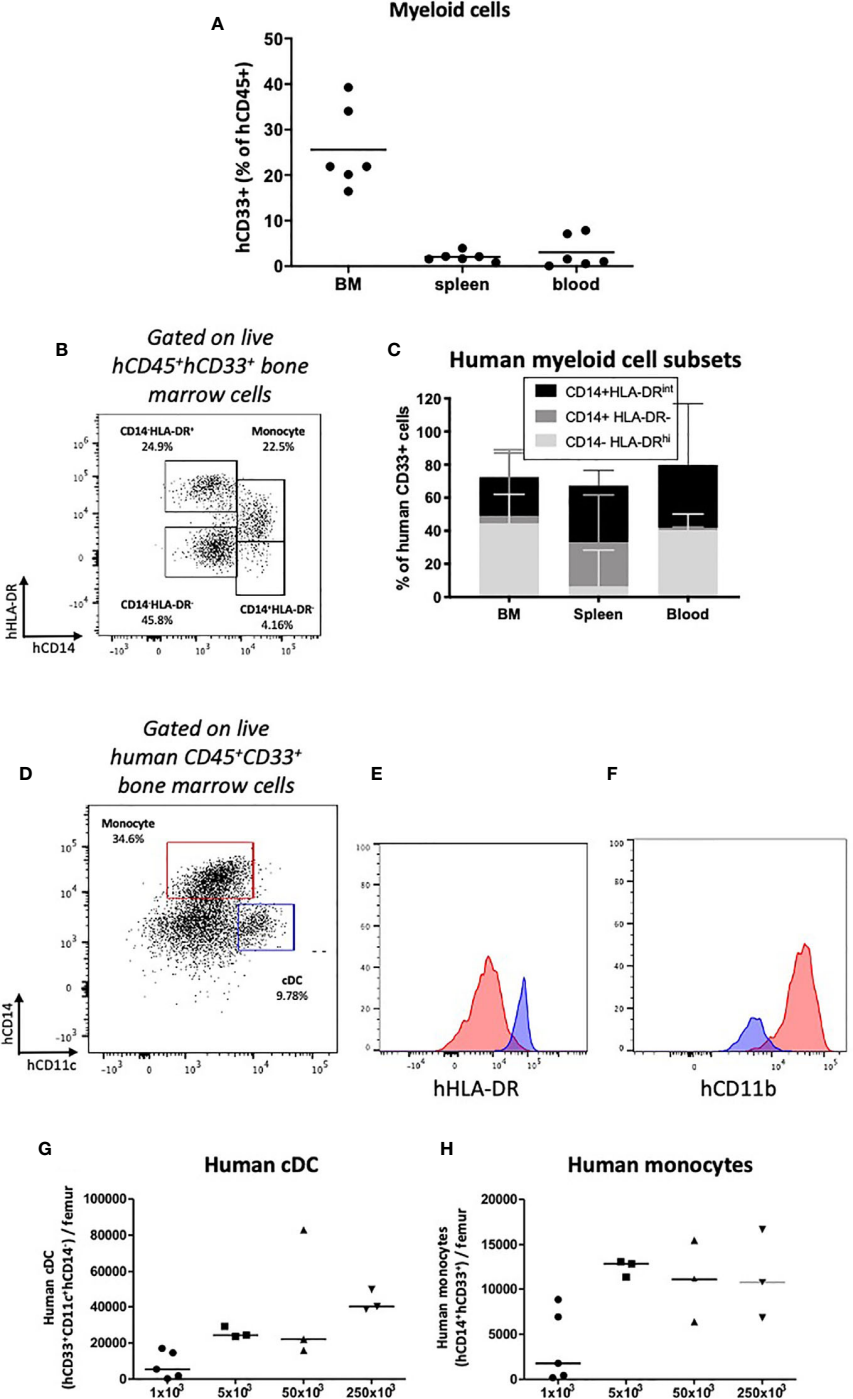
Human myeloid cell engraftment in HSPC-NBSGW mice. **(A)** Frequencies of human CD33^+^ myeloid cells in the bone marrow, spleen and peripheral blood of HSPC-NBSGW mice 20-22 weeks after cell injection. **(B)** Representative flow cytometric plot and **(C)** corresponding frequencies of myeloid subsets based on CD14 and HLA-DR expression. **(D)** Representative flow cytometry plot and **(E, F)** corresponding histograms demonstrating human conventional dendritic cells (cDCs) (blue; hCD11c^+^hCD14^-^hCD33^+^) and human monocytes/macrophages (red; hCD14^+^hCD33^+^) in the bone marrow of HSPC-NBSGW mice (50x10^3^ dose shown). Histograms demonstrate high HLA-DR expression and low CD11b expression in cDCs (blue histograms) as compared with monocytes (red histograms). **(G)** Numbers of human cDCs (hCD11c+hCD14-hCD33+) **(H)** and human monocytes/macrophages (hCD14^+^hCD33^+^) in the bone marrow of one femur in mice receiving different numbers of hUCB CD133^+^ HSPCs. Bars indicate median values **(A, G, H)** and median + IQR **(C)**.

Developing humanized mouse models capable of reconstituting cells of the innate and adaptive immune systems and long-term survival is difficult to achieve, especially in the absence of irradiation and engineered or exogenous cytokine expression. Having identified engraftment of human CD33^+^ myeloid cells (**Figure 3A**; **Supplementary Table 2**; **Supplementary Figure 2**), we assessed subtype reconstitution (**Figure 6**). At all doses of HSPCs transplanted, myeloid cells engrafted with the greatest frequency in the bone marrow (**Figure 6A**; **Supplementary Table 2**). Within the bone marrow and spleen we identified populations of HLA-DR^hi^, HLA-DR^int^ and HLA-DR^-^ cells (**Figures 6B, C**). The CD33^+^HLA-DR^hi^ subset, which includes CD11c^+^ conventional dendritic cells (cDCs), formed the most common subtype in the bone marrow (**Figures 6B–G**; **Supplementary Figures 5A, B**). As similarly described in human bone marrow, these cDCs expressed CD33, high levels of CD11c and HLA-DR, and were CD14^-^ (37) and CD11b^lo/-^ aiding the distinction from monocyte/macrophage lineage cells (**Figures 6D–F**). The HLA-DR^int^ fraction, which includes CD14-expressing mature monocytes/macrophages is found predominantly in the blood where it forms approximately half of all myeloid cells (**Figures 6B–F** and **H**). A less mature CD14^+^HLA-DR^-^ population, which may include myeloid-derived suppressor cells is also present, especially in the spleen (**Figure 6C**). Together, these data demonstrate successful engraftment and reconstitution of phenotypically distinct subsets of innate myeloid cells, which express molecules of antigen presentation.

### Phagocytosis-Dependent Impairment in Peripheral Engraftment of Human Erythrocytes

**Figure 7:**
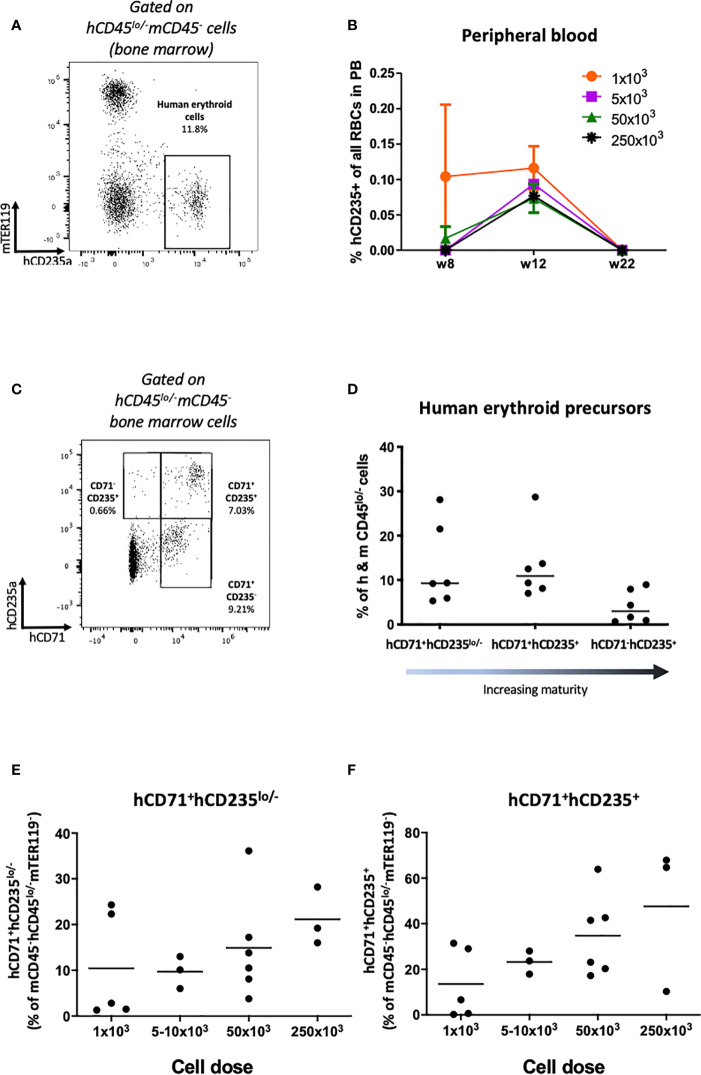
Human erythrocyte reconstitution in HSPC-NBSGW mice. **(A)** Representative flow cytometric analysis demonstrating the identification of hCD45^lo/-^mCD45^-^hCD235a^+^mTER119^-^ human erythroid cells in the bone marrow. **(B)** Frequency of hCD235^+^ cells in the peripheral blood at different time points following injection with increasing numbers of hUCB CD133^+^ HSPCs (mean ± SD shown). **(C)** Representative flow cytometric analysis and **(D)** corresponding frequencies of hCD71^+^hCD235^lo/-^, hCD71^+^hCD235^+^ and hCD71^-^hCD235^+^ erythroid precursors (in ascending order of maturity) in the bone marrow (BM) 20-22 weeks after humanization (50x10^3^ dose shown). **(E)** Frequency of hCD71^+^hCD235^-/lo^ and **(F)** hCD71^+^hCD235^+^ erythroid precursors in the bone marrow (BM) following humanization with increasing numbers of hUCB CD133^+^ HSPCs. Red symbols – female mice, blue symbols – male mice. Bars indicate median values.

**Figure 8:**
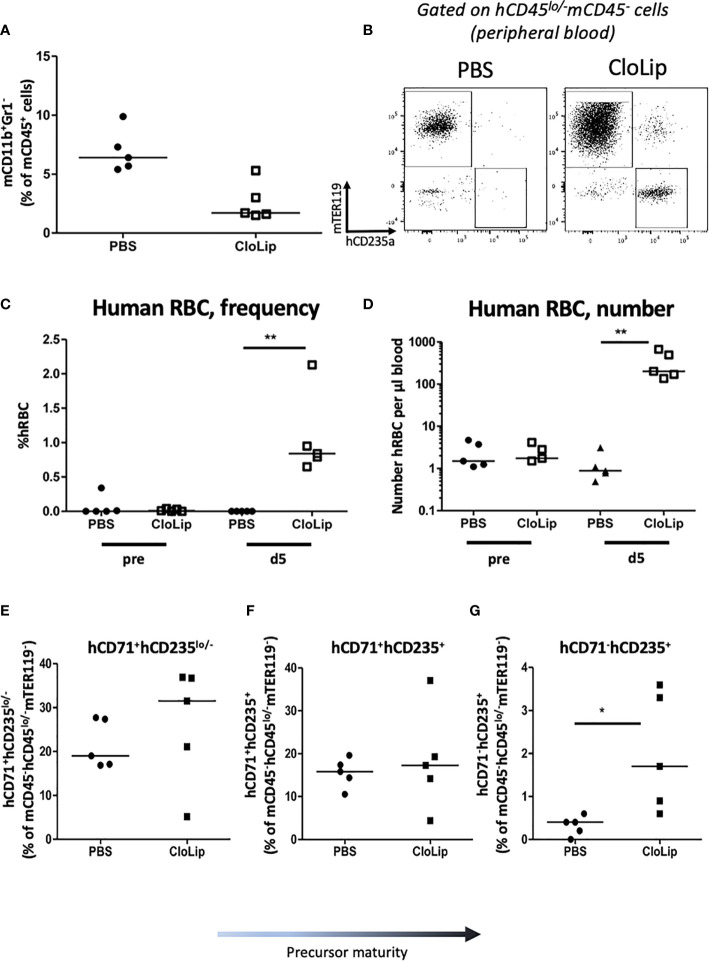
Effect of murine phagocyte clearance on human erythrocyte reconstitution HSPC-NBSGW mice. **(A–G)** HSPC-NBSGW mice injected with 50x10^3^ hUCB CD34^+^ cells 11 weeks earlier were injected with clodronate liposomes (CloLip) or PBS (control) and analyzed over 6 days. **(A)** Frequency of mouse CD11b^+^Gr1^-^ cells in the blood 5 days after clodronate liposome (CloLip) treatment. **(B)** Representative flow cytometric analysis of human CD235^+^ RBCs in the blood 5 days after CloLip treatment. **(C)** frequency and **(D)** absolute number of human RBCs (hCD235^+^) in the peripheral blood 5 days after CloLip treatment. **(E)** Frequencies of hCD71^+^hCD235^-/lo^, **(F)** hCD71^+^hCD235^+^ and **(G)** hCD71^-^hCD235^+^ in the bone marrow (BM) 6 days after CloLip/PBS treatment. Symbols represent individual mice. Bars represent median values **(A)**–**(C–G)**; Statistical significance was assessed using the Mann Whitney test (*p<0.05, **p<0.01).

Next we investigated engraftment of erythrocytes. In HSPC-NBSGW mice, Glycophorin A analysis (CD235a) demonstrated dose-dependent engraftment of erythroid cells within the bone marrow, however only a small, transient population of RBCs were found in the peripheral blood (**Figures 7A, B**, **Supplementary Figure 6A**). To determine whether absence from the periphery results from defective erythroid lineage differentiation, we assessed erythroid cell maturity in the bone marrow (**Figures 7C–F**). In addition to a small population of mature erythrocytes (**Figures 7C, D**; **Supplementary Figure 6A**), dose-dependent frequencies of erythroid precursors at various stages of development were identified, with no significant discrepancies noted between male and female mice (**Figures 7E, F**; **Supplementary Figures 6B–D**). To identify whether the lack of peripheral reconstitution results instead from phagocytosis of human erythroid cells by mouse phagocytes, we assessed survival following phagocyte depletion (**Figures 8A–G**). Using a previously reported technique (27), clodronate liposomes (CloLip) were injected intravenously 11 weeks after humanization, successfully reducing the frequencies of monocytes in the peripheral blood (**Figure 8A**). This was associated with increased numbers of mature erythrocytes in the blood (168-fold compared with control; 335 ± 237 vs 2 ± 1.6 per μl of blood) (**Figures 8B–D**), suggesting that phagocytosis contributes at least in part to the impaired reconstitution of human erythroid cells in this model. While there was no significant difference in the frequencies of early erythroid precursors in the bone marrow following phagocyte clearance, as expected, an increase in reticulocytes and mature erythrocytes was seen (19% (16-27.7%) vs 31.5% (5.2-36.9%) as median (with range), p<0.05) (**Figures 8E–G**).

## Discussion

Here we show robust long-term multilineage reconstitution of human hematopoeitic cells in NBSGW mice. We also show that the numbers of circulating RBCs can be increased in NBSGW mice following mouse macrophage clearance, enhancing the capacity to study disorders of human erythropoiesis in the context of complete human lymphoid development.

For optimal human HSC engraftment, there must be a permissive bone marrow niche in favor of human hematopoiesis. This has been achieved through 1) mutation of critical murine growth factor receptors such as the c-kit receptor (W41/Wv alleles) in NSGW41, NSGWv/+, NSGWv, NBSGW, SRG-W41 and BRgWv mice, 2) direct treatment with an anti-c-Kit receptor antibody, 3) provision of exogenous human cytokines, 4) knock-in of human cytokine genes (e.g. SCF, TPO, GM-CSF, M-CSF, IL-3 in NSG-SGM3/NSGS, MSTRG and MISTRG mice) (4, 5, 8, 36, 38–43), and 5) transplant of hematopoietic niches (e.g. human mesenchymal stromal cells, or thymus) with or without expression of human cytokine transgenes [e.g. NSG-SGM3-BLT mice (44)]. NSGW41, BRgWv, NBSGW and SRG-W41 mice are reported to show robust human HSPC engraftment in the absence of irradiation and this is coupled with improved human bone marrow erythropoiesis compared to irradiated NSG mice (4, 36, 38–40, 43–45). Our results extend these and our own previous findings (29) by demonstrating consistent engraftment and human leucocyte reconstitution following intravenous transplantation of as few as 10x10^3^ CD133^+^ HSPCs in adult nonirradiated NBSGW recipients over the extended period of 20-22 weeks, regardless of sex and without the requirement to co-transplant other human hematopoietic niche tissues. To our knowledge this is the lowest hUCB HSPC dose reported to achieve such high BM (75.5%), splenic (73.5%) and peripheral blood (29%) engraftment at this late a time point. It is also notable, that in this model, leucocyte subset reconstitution approximated more closely to human peripheral blood populations (46) than previously described in humanized NBSGW mice (5). The capacity to achieve robust reconstitution in adult mice is particularly attractive due to the ease of intravenous injection compared with neonates and the potential simplicity of injecting fresh cells, with the flexibility of not requiring timed breeding.

Importantly, we have also demonstrated that development and maturation of human lymphocytes and myeloid cells occurs within this model, producing a humanized mouse that represents a more complete human immune system. Reconstitution of both T and B lymphocytes is important for studies of vaccination and infection immunity, enabling incorporation of humoral and cellular components of the human immune system. Additionally, as effective antibody class switching is dependent on T cell help, humanized mice supporting robust T and B cell reconstitution can allow studies on the development of protective humoral immunity in various infectious diseases.

hUCB HSPC-humanized mice typically support engraftment of human B cells at high frequencies (47). In several models, these develop quite normally in the bone marrow yet demonstrate features of developmental block (48), manifesting defective peripheral maturation and humoral responses (1, 49). We have demonstrated development of a large population of mature B cells in the spleens and peripheral blood of HSPC-NBSGW mice. Since only mature B cells are capable of carrying out effective antigen presentation and effector function, this is likely to be a distinguishing feature of this model. We further demonstrate the frequency of class-switched memory B cells to increase following exposure to allogeneic human skin transplants. This feature suggests the functional capacity for HSPC-NBSGW B cells to respond to an immune challenge and may represent an important step in fields such as transplantation research, where improvements enhanced human T- and B function may enable more accurate reflections of human alloresponses. As the detrimental effects of donor-specific antibodies and the tolerogenic effects of regulatory B cells are increasingly becoming focal points of attention in transplantation, models such as this may add functional humoral immunity to the predominantly T cell biased PBMC-based humanized models.

The re-creation of cellular immune responses is a fundamental requirement of HIS mice. Following humanization with CD133^+^ hUCB HSPCs, we demonstrate peripheral reconstitution of the T cell subsets in proportions that more accurately reflect the human blood profile than can be achieved following humanization with PBMCs. It should be noted, that while PBMC-driven HIS models have been very useful in studying T cell-driven immune responses (50), cellular therapies (51) and biologics (52), disproportionately large T cell reconstitution in HIS mice generated using PBMCs (particularly Tem cells) risks the development of xenogeneic GvHD and underrepresentation of the global immunological complexity.

Within the thymus we identified a humanized thymic cell microenvironment with a sizeable fraction of CD4^+^CD8^+^ (Double positive, DP) human thymocytes, similar to that seen in thymus biopsies from human infants (36). Identification of T cells in the peripheral blood only after 12 weeks in the earliest instances, suggests *de novo* T cell development rather than proliferation of transplanted mature hUCB MNCs.

In our experience, engraftment of 50x10^3^ human HSPCs produces rapid, robust and reliable reconstitution, supporting utility of this dose for a broad range of *in vivo* experiments. A recognized shortcoming of utilising human cord blood in mouse experiments - which we also experienced - is the limited number of mice that can be engrafted from a single UCB unit when high numbers of HSPCs are used per mouse. It is our view that experimental biases that result from low numbers of donor replicates may be overcome if more mice can be robustly engrafted (i.e. >20% peripheral blood chimerism) using smaller UCB fractions (i.e. <50x103 HSPCs). We show that this is possible and feel that where investigators may find benefit in expanding the utility of a single UCB unit, a longer period of engraftment may be an acceptable compromise, particularly considering the reported longevity of humanized NBSGW mice (survival ≥ 36 weeks post-humanization (6)).

As efforts continue to broaden the use of humanized small animal models for the *in vivo* study of human hematopoiesis, immunology, cancer and infection, technically uncomplicated models achieving ever-closer functional human hematopoiesis such as the one reported here represent an important practical next step.

## Data Availability Statement

The raw data supporting the conclusions of this article will be made available by the authors, without undue reservation.

## Ethics Statement

hUCB was collected from the John Radcliffe Hospital, Oxford, UK or provided *via* the NHS Cord Blood Bank, London and used with informed, written pre-consent and ethical approval from the South Central Oxford C and Berkshire Ethical Committees (# 15/SC/0027) and the Oxfordshire Research Ethics Committee B (#07/H0605/130), in accordance with the Helsinki Declaration of 1975, as revised in 2008. Written informed consent to participate in this study was provided by the participants’ legal guardian/next of kin. All mouse experiments were performed using protocols approved by the Committee on Animal Care and Ethical Review at the University of Oxford and in accordance with the UK Animals (Scientific Procedures) Act 1986 and under PPL number P8869535A.

## Author Contributions

GA: conception and design, collection and assembly of data, data analysis and interpretation, manuscript writing, and final approval of manuscript. PH: conception and design, collection and/or assembly of data, data analysis and interpretation, manuscript writing, and final approval of manuscript. MU: collection and assembly of data, manuscript writing, and final approval of manuscript. IR: conception and design, financial support, data analysis and interpretation, and final approval of manuscript. JH: conception and design, collection and assembly of data, data analysis and interpretation, financial support, manuscript writing, and final approval of manuscript. SW: conception and design, financial support, data analysis and interpretation, manuscript writing, and final approval of manuscript. FI: conception and design, financial support, collection and assembly of data, data analysis and interpretation, manuscript writing, and final approval of manuscript. All authors contributed to the article and approved the submitted version.

## Funding

MRC Discovery Award: Weatherall Institute of Molecular Medicine grant no. MC_PC_15069 (lead investigator: Professor D. Higgs). Kidney Research UK Senior Fellowship (JH) (SF1/2014), Wellcome Trust CRCD Fellowship (FI) Clarendon Scholarship (GA).

## Conflict of Interest

The authors declare that the research was conducted in the absence of any commercial or financial relationships that could be construed as a potential conflict of interest.
