# Supplementary figures and images for: Development of LT-HSC-Reconstituted Non-Irradiated NBSGW Mice for the Study of Human Hematopoiesis In Vivo
Source: Front Immunol. 2021 Mar 25;12:642198. doi: 10.3389/fimmu.2021.642198 (PMC8044770; doi:10.3389/fimmu.2021.642198)

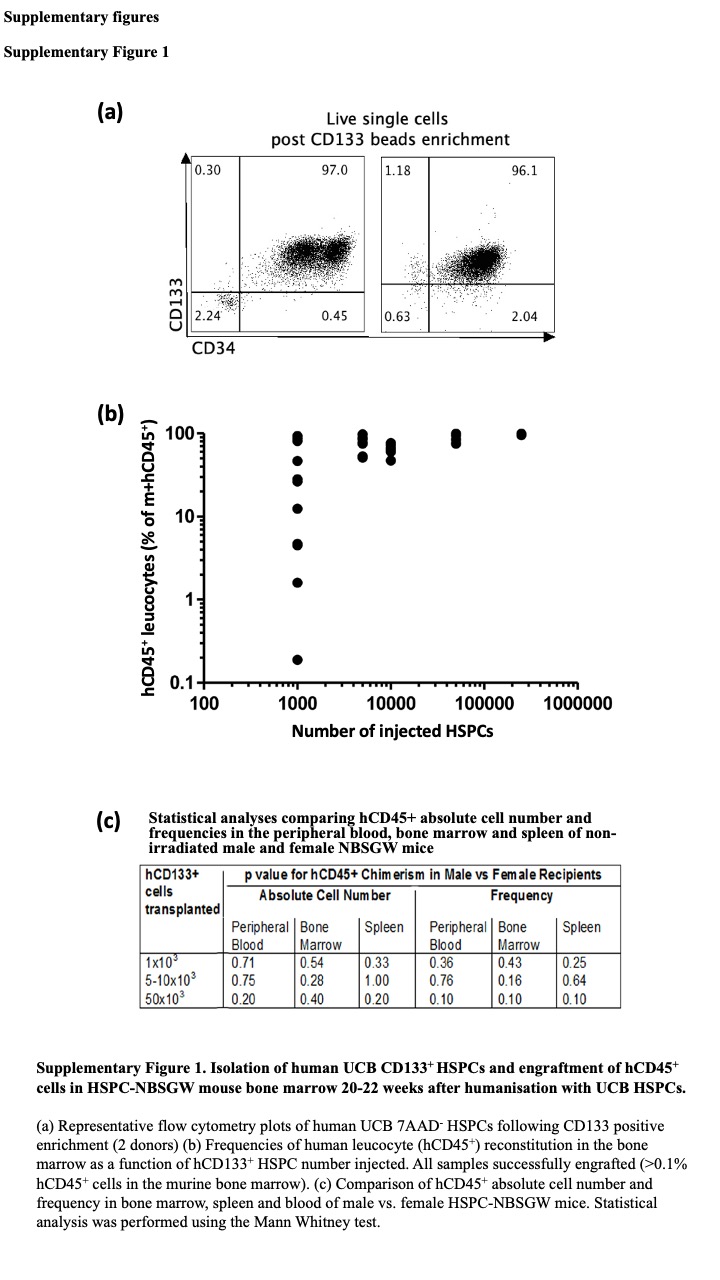

Supplement: Supplementary file 1 [file Image_1.jpeg]

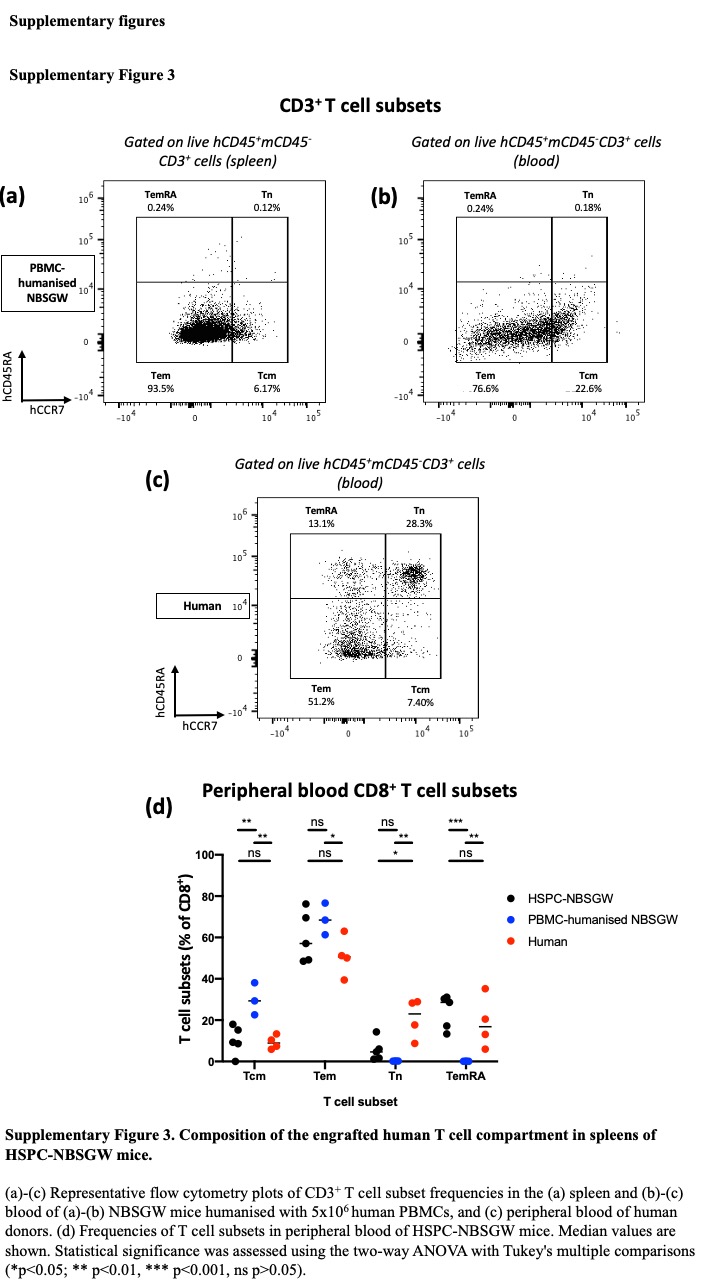

Supplement: Supplementary file 3 [file Image_3.jpeg]

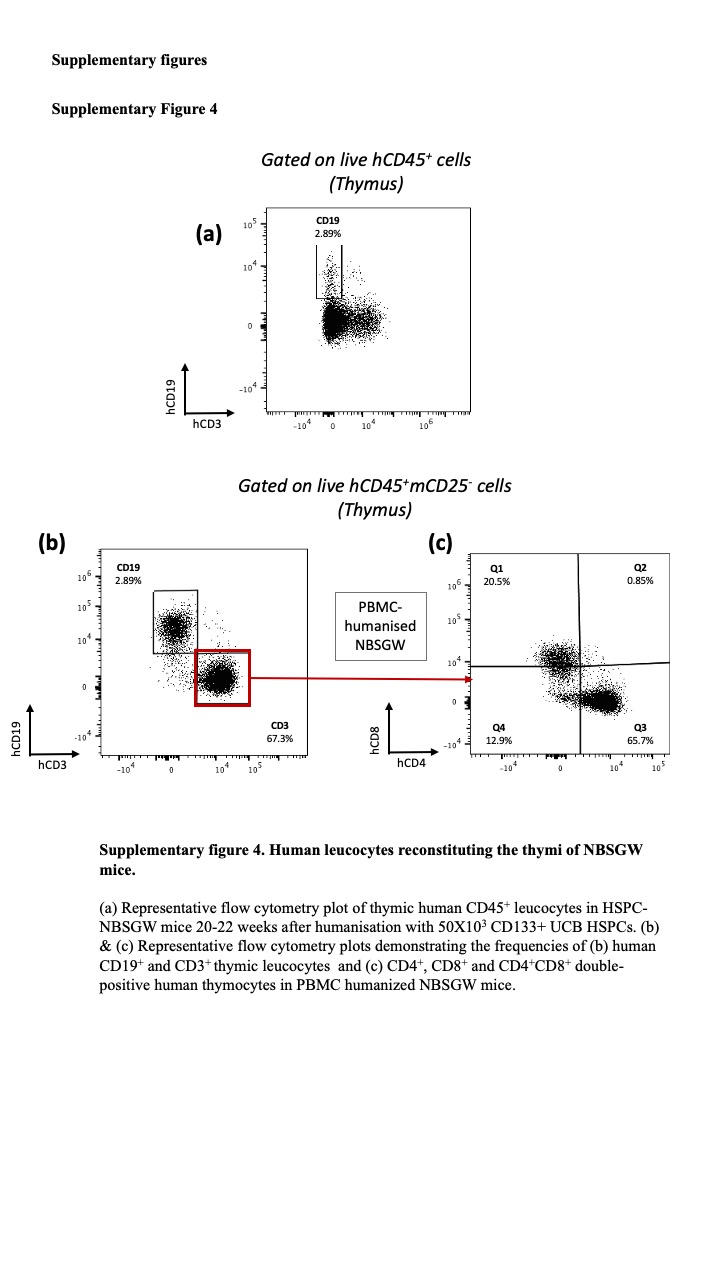

Supplement: Supplementary file 4 [file Image_4.jpeg]

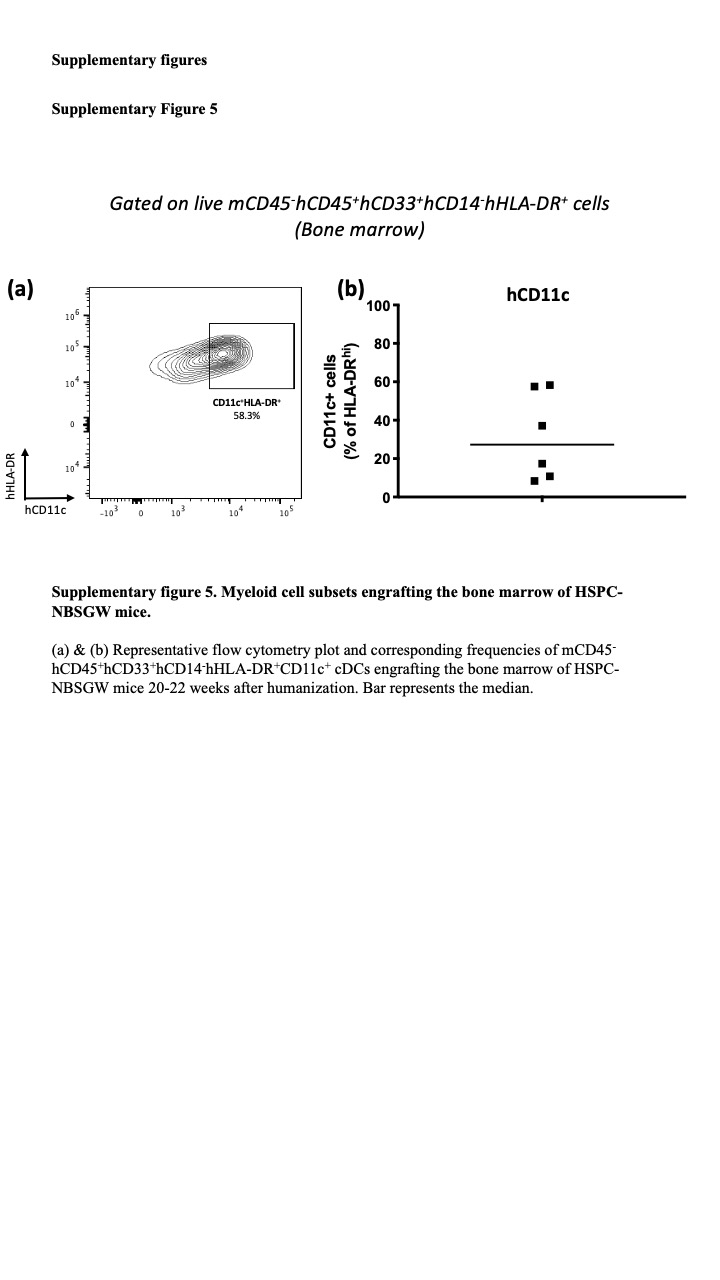

Supplement: Supplementary file 5 [file Image_5.jpeg]
