# Supplementary material for: Development of LT-HSC-Reconstituted Non-Irradiated NBSGW Mice for the Study of Human Hematopoiesis In Vivo
Source: Front Immunol. 2021 Mar 25;12:642198. doi: 10.3389/fimmu.2021.642198 (PMC8044770; doi:10.3389/fimmu.2021.642198)
Supplement: Supplementary file 2 [file Image_2.pdf]

Supplementary figures

Supplementary Figure 2

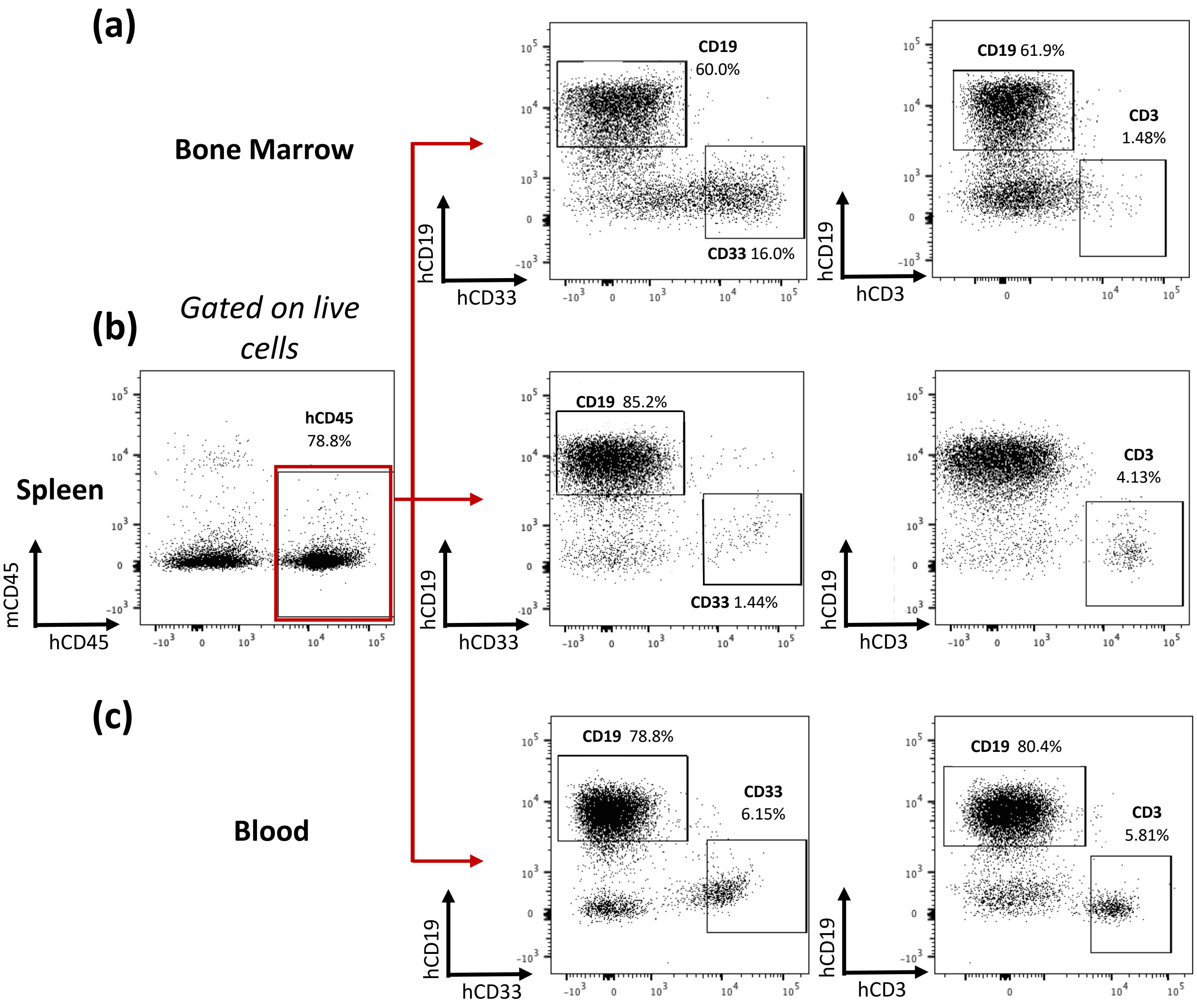

Supplementary figures

Supplementary Figure 2 cont'd

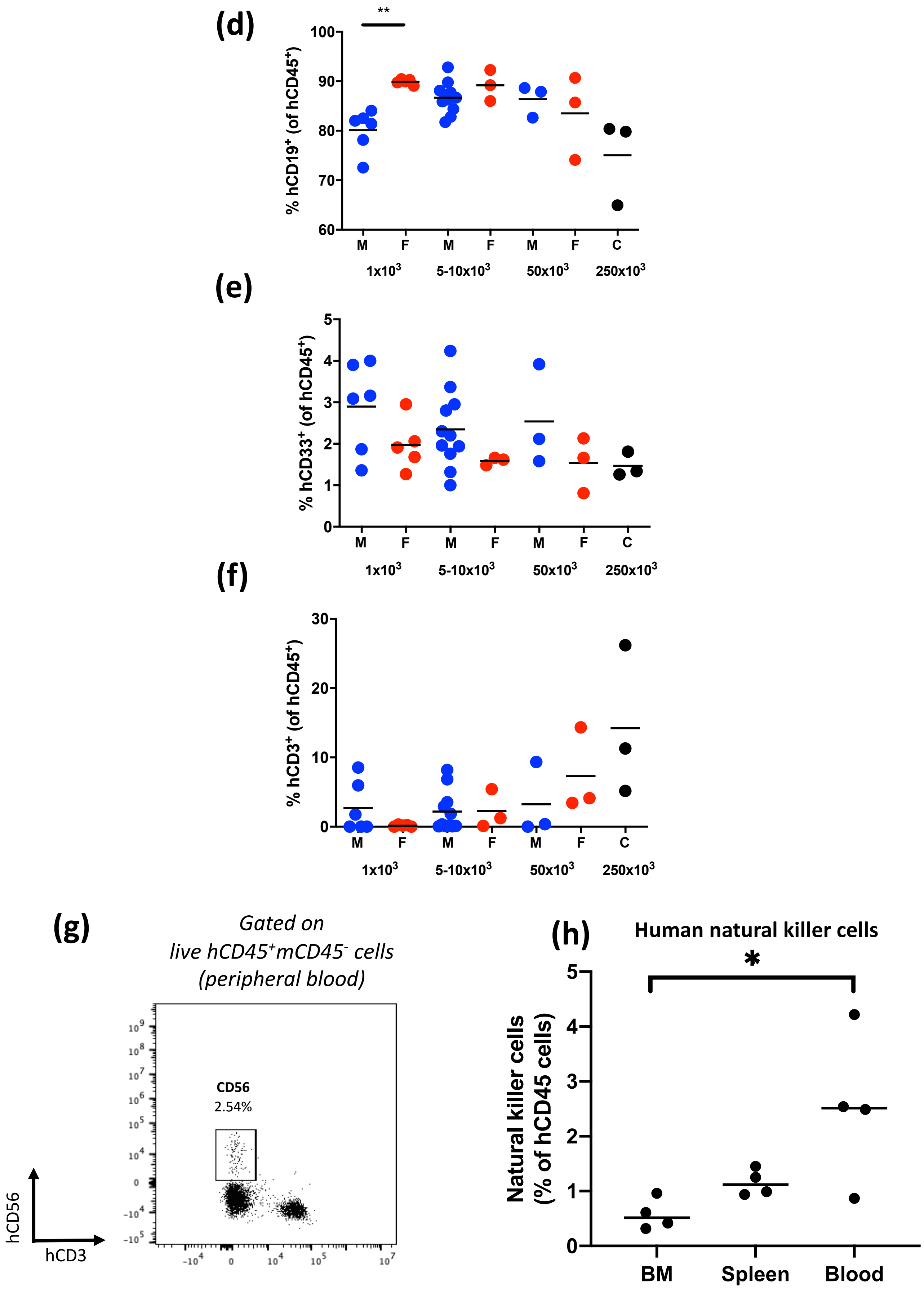

Supplementary figure 2. Engraftment of leucocytes in HSPC-NBSGW mice.

(a)-(c) Representative flow cytometry plots of human B (hCD19$^+$) versus myeloid (hCD33$^+$) or T (CD3$^+$) cell reconstitution in the (a) bone marrow, (b) spleen and (c) blood of HSPC-NBSGW mice 20-22 weeks after humanisation. (d)-(f) Frequencies of human (d) B cells (hCD19$^+$), (e) myeloid cells (hCD33$^+$), and (f) T cells (hCD3$^+$) in the spleens of male (blue symbols) and female (red symbols) mice reconstituted with different numbers of HSPCs as indicated. (g) Representative flow cytometry plot and (h) corresponding frequencies of hCD45$^+$mCD45$^-$CD56$^+$ natural killer cells reconstituting HSPC-NBSGW bone marrow, spleens and peripheral blood 20-22 weeks after humanisation (only 50x10^3 dose assessed, as shown). Bars indicate median values. Statistical significance was assessed using the unpaired Mann-Whitney test ((d)-(f)) and the Ordinary one-way ANOVA with Holm-Šídák's multiple comparisons test (h) (*$p<0.05$; ** $p<0.01$).

Supplementary figures

Supplementary Figure 2 cont'd

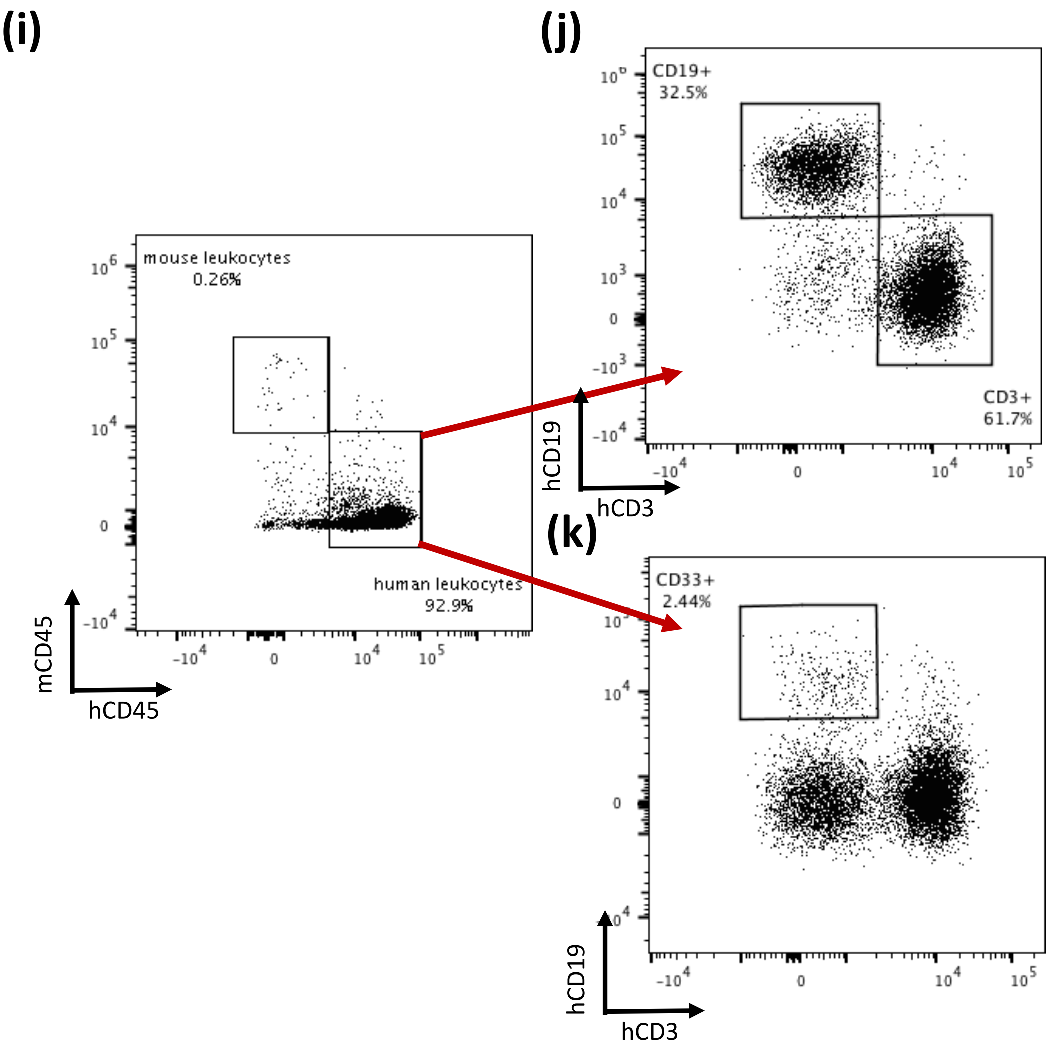

Supplementary figure 2. Engraftment of leucocytes in HSPC-NBSGW mice.

(a)-(c) Representative flow cytometry plots of human B ($hCD19^{+}$) versus myeloid ($hCD33^{+}$) or T ($CD3^{+}$) cell reconstitution in the (a) bone marrow, (b) spleen and (c) blood of HSPC-NBSGW mice 20-22 weeks after humanisation. (d)-(f) Frequencies of human (d) B cells ($hCD19^{+}$), (e) myeloid cells ($hCD33^{+}$), and (f) T cells ($hCD3^{+}$) in the spleens of male (blue symbols) and female (red symbols) mice reconstituted with different numbers of HSPCs as indicated. (g) Representative flow cytometry plot and (h) corresponding frequencies of $hCD45^{+}mCD45^{-}CD56^{+}$ natural killer cells reconstituting HSPC-NBSGW bone marrow, spleens and peripheral blood 20-22 weeks after humanisation (only $50x10^{3}$ dose assessed, as shown). (i)-(k) Representative flow cytometry plots of human leukocyte (i), B cell, T cell (j) and myeloid cell (k) reconstitution in spleens of HSPC-NBSGW mice transplanted with split-thickness human skin allografts 59 days earlier. Bars indicate median values. Statistical significance was assessed using the unpaired Mann-Whitney test ((d)-(f)) and the Ordinary one-way ANOVA with Holm-Šídák's multiple comparisons test (h) (*p<0.05; ** p<0.01).
