# Supplementary material for: Development of LT-HSC-Reconstituted Non-Irradiated NBSGW Mice for the Study of Human Hematopoiesis In Vivo
Source: Front Immunol. 2021 Mar 25;12:642198. doi: 10.3389/fimmu.2021.642198 (PMC8044770; doi:10.3389/fimmu.2021.642198)
Supplement: Supplementary file 6 [file Image_6.pdf]

Supplementary figures

Supplementary Figure 6

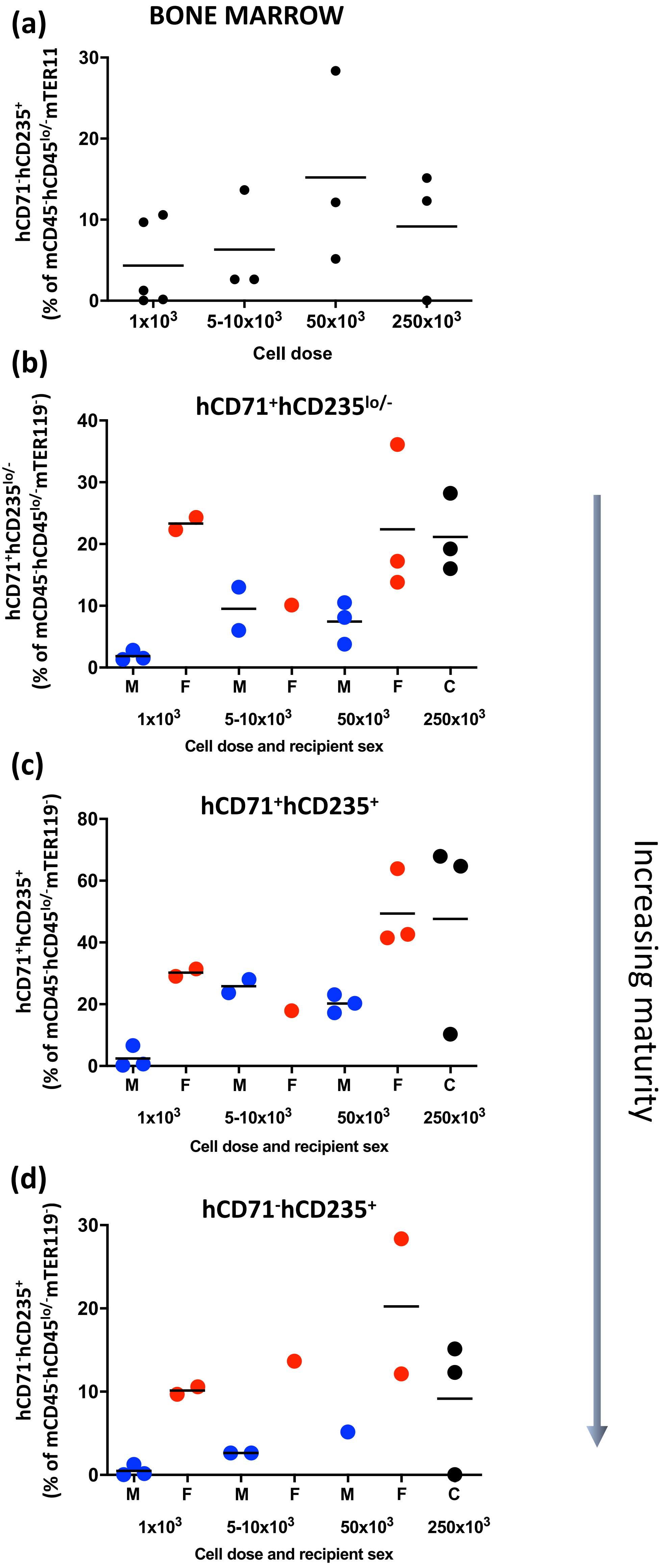

Supplementary figure 6. Human erythrocytes engraft the bone marrow in HSPC-NBSGW mice.

Frequencies of (a) hCD71⁻hCD235⁺ erythroid cells reconstituting the bone marrow of HSPC-NBSGW mice 20-22 weeks after humanisation with increasing numbers of hUCB CD133⁺ HSPCs. (b)-(d) Comparison of precursor engraftment in male (blue symbols) and female (red symbols) mice. (Bars represent median values)
