# Supplementary material for: Development of LT-HSC-Reconstituted Non-Irradiated NBSGW Mice for the Study of Human Hematopoiesis In Vivo
Source: Front Immunol. 2021 Mar 25;12:642198. doi: 10.3389/fimmu.2021.642198 (PMC8044770; doi:10.3389/fimmu.2021.642198)
Supplement: Supplementary file 7 [file Table_1.docx]

**Supplementary Table 1. List of antibodies for flow cytometry**

| **Antigen and fluorochrome** | **Supplier (cat number)** | **Clone** |
| --- | --- | --- |
| Anti-human CD45RA FITC | BioLegend Cat# 304106, RRID:AB_314410 | HI100 |
| Anti-human CD27 FITC | BD Biosciences Cat# 555440, RRID:AB_395833 | M-T271 |
| Anti-human Lineage FITC  (anti-CD3, CD14, CD16, CD19,  CD20, CD56) | BD Biosciences Cat# 340546, RRID:AB_400053 | SK7 (CD3), 3G8  (CD16), SJ25C1  (CD19), L27 (CD20),  MφP9 (CD14),  NCAM16.2 (CD56) |
| Anti-human CD11b FITC | Thermo Fisher Scientific Cat# 11-0118-42, RRID:AB_1582242 | ICRF44 |
| Anti-human CD3 FITC | BioLegend Cat# 300406, RRID:AB_314060 | UCHT1 |
| Anti-human CD14 FITC | BioLegend Cat# 325604, RRID:AB_830677 | HCD14 |
| Anti-human CD19 FITC | BioLegend Cat# 302206, RRID:AB_314236 | HIB19 |
| Anti-human CD8 PE | BD Biosciences Cat# 555635, RRID:AB_395997 | HIT8a |
| Anti-human CD19 PE | BioLegend Cat# 302208, RRID:AB_314238 | HIB19 |
| Anti-human CD34 PE | Thermo Fisher Scientific Cat# 12-0349-42, RRID:AB_1548680 | 4H11 |
| Anti-human CD61 PE | BioLegend Cat# 336406, RRID:AB_2128752 | VI-PL2 |
| Anti-human CD14 PE | Thermo Fisher Scientific Cat# 12-0149-42, RRID:AB_10598367 | 61D3 |
| Anti-human HLA-DR ECD | Beckman Coulter Cat# IM3636, RRID:AB_10643231 | Immu-357 |
| Anti-human CD3 PE-Cy7 | BD Biosciences Cat# 557851, RRID:AB_396896 | SK7 |
| Anti-human IgD PE-Cy7 | BD Biosciences Cat# 561314, RRID:AB_10642457 | IA6-2 |
| Anti-human CD33 PE-Cy7 | Thermo Fisher Scientific Cat# 25-0338-42, RRID:AB_1907380 | WM-53 |
| Anti-human CD71 PE-Cy7 | BioLegend Cat# 334112, RRID:AB_2563119 | CY1G4 |
| Anti-human CCR7 APC | BioLegend Cat# 353213, RRID:AB_10915474 | G043H7 |
| Anti-human CD10 APC | BioLegend Cat# 312210, RRID:AB_314921 | HI10a |
| Anti-human CD38 APC | Thermo Fisher Scientific Cat# 17-0389-41, RRID:AB_1834354 | HIT2 |
| Anti-human CD45 APC | Thermo Fisher Scientific Cat# MHCD4505, RRID:AB_10372216 | HI30 |
| Anti-humanCD235a (Gly A)  APC | Thermo Fisher Scientific Cat# 17-9987-42, RRID:AB_2043823 | HIR2 (GA-R2) |
| Anti-human CD11c APC | BD Biosciences Cat# 559877, RRID:AB_398680 | B-ly6 |
| Anti-human CD4 APC | BD Biosciences Cat# 555349, RRID:AB_398593 | RPA-T4 |
| Anti-human CD45 APC/Fire 750 | BioLegend Cat# 368518, RRID:AB_2616705 | 2D1 |
| Anti-human CD3 APC-eFluor  780 | Thermo Fisher Scientific Cat# 47-0038-42, RRID:AB_1272042 | UCHT1 |
| Anti-mouse Ter119 FITC | Thermo Fisher Scientific Cat# 11-5921-82, RRID:AB_465311 | TER-119 |
| Anti-mouse CD41 PerCP/Cy5.5 | BioLegend Cat# 133918, RRID:AB_2563500 | MWReg30 |
| Anti-mouse CD11b PE-Cy7 | Thermo Fisher Scientific Cat# 25-0112, RRID:AB_2314132 | M1/70 |
| Anti-mouse CD45 eFluor450 | Thermo Fisher Scientific Cat# 48-0451-82, RRID:AB_1518806 | 30-F11 |
| Anti-mouse Gr-1 APC | Thermo Fisher Scientific Cat# 17-5931-81, RRID:AB_469475 | RB6-8C5 |
| 7-AAD | eBioscience (00-6993-50) | N/A |
