# Supplementary material for: Development of LT-HSC-Reconstituted Non-Irradiated NBSGW Mice for the Study of Human Hematopoiesis In Vivo
Source: Front Immunol. 2021 Mar 25;12:642198. doi: 10.3389/fimmu.2021.642198 (PMC8044770; doi:10.3389/fimmu.2021.642198)
Supplement: Supplementary file 8 [file Table_2.pdf]

Supplementary tables

Supplementary table 2. Frequencies of human leucocyte populations in the spleen, bone marrow and peripheral blood 20-22 weeks post-transplantation

		$1x10^3$ (n=11)	$5x10^3$ (n=7)	$10x10^3$ (n=7)	$50x10^3$ (n=7)	$250x10^3$ (n=3)
**spleen**						
**% hCD45+**	mean (SD)	32.0 (39.4)	73.5 (25.4)	79.0 (10.7)	93.6 (5.3)	98.5 (0.9)
**% CD19+**	mean (SD)	84.6 (5.9)	86.9 (3.2)	87.5 (3.4)	85.0 (6.0)	75.1 (8.7)
**% CD33+**	mean (SD)	2.5 (1.0)	2.2 (1.2)	2.2 (0.6)	2.0 (1.0)	1.5 (0.3)
**% CD3+**	mean (SD)	1.5 (2.9)	1.8 (3.0)	2.6 (2.7)	5.3 (5.6)	14.2 (10.8)
**BM**						
**% hCD45+**	mean (SD)	35.0 (36.2)	75.5 (17.6)	64.6 (9.5)	87.8 (11.0)	97.4 (1.9)
**% CD19+**	mean (SD)	72.1 (17.0)	69.0 (22.5)	81.4 (9.3)	54.5 (18.8)	36.7 (3.9)
**% CD33+**	mean (SD)	7.9 (11.0)	11.7 (14.2)	9.0 (11.5)	25.6 (8.9)	26.4 (6.8)
**% CD3+**	mean (SD)	0.8 (1.1)	1.0 (1.6)	0.9 (1.5)	1.8 (2.0)	8.5 (6.7)
**blood**						
**% hCD45+**	mean (SD)	12.1 (24.7)	29.0 (30.0)	28.1 (12.8)	50.6 (31.7)	80.0 (14.2)
**% CD19+**	mean (SD)	50.4 (41.1)	76.6 (16.7)	85.2 (5.5)	77.1 (7.9)	51.5 (14.6)
**% CD33+**	mean (SD)	2.7 (3.8)	8.5 (9.9)	1.1 (1.2)	3.0 (3.5)	8.2 (3.3)
**% CD3+**	mean (SD)	0.4 (0.9)	1.8 (4.4)	2.8 (3.2)	7.5 (6.2)	18.4 (8.1)

$hCD45^+$ as % of $h+mCD45^+$, $hCD19^+$, $hCD33^+$, $hCD3^+$ as % of $hCD45^+$
