# Supplementary material for: Development of LT-HSC-Reconstituted Non-Irradiated NBSGW Mice for the Study of Human Hematopoiesis In Vivo
Source: Front Immunol. 2021 Mar 25;12:642198. doi: 10.3389/fimmu.2021.642198 (PMC8044770; doi:10.3389/fimmu.2021.642198)
Supplement: Supplementary file 9 [file Table_3.pdf]

Supplementary tables

Supplementary table 3. Absolute cell numbers of human leucocyte populations in the spleen, bone marrow and peripheral blood.

		$1x10^3$ (n=11)	$5x10^3$ (n=7)	$10x10^3$ (n=7)	$50x10^3$ (n=7)	$250x10^3$ (n=3)
**spleen**						
**hCD45+**	mean (SD)	873697 (1597728)	2665263 (1927842)	1762045 (1216759)	5400707 (3069853)	9575582 (3858274)
**CD19+**	mean (SD)	781801 (1438353)	2323283 (1684014)	1514711 (969695)	4488147 (2425023)	7308951 (3487852)
**CD33+**	mean (SD)	15813 (25997)	46023 (25698)	39518 (30484)	97262 (64989)	135981 (39324)
**CD3+**	mean (SD)	3357 (4736)	74811 (135535)	62044 (106323)	399407 (464568)	1290485 (801811)
**BM**						
**hCD45+**	mean (SD)	266284 (378622)	634043 (521090)	404888 (381261)	852015 (481297)	821951 (304156)
**CD19+**	mean (SD)	173734 (239825)	364826 (184394)	320377 (291311)	409470 (172648)	296236 (99304)
**CD33+**	mean (SD)	43122 (80370)	119812 (178873)	55818 (86370)	219477 (143491)	229085 (121670)
**CD3+**	mean (SD)	2178 (4208)	8083 (10125)	3869 (4519)	17083 (17397)	56467 (28179)
**blood**						
**hCD45+**	mean (SD)	16.54 (44.70)	43.57 (57.03)	17.88 (11.69)	83.61 (91.70)	92.62 (52.84)
**CD19+**	mean (SD)	14.14 (38.64)	33.88 (32.87)	15.15 (10.06)	64.00 (70.97)	43.11 (18.58)
**CD33+**	mean (SD)	0.28 (0.56)	6.30 (13.97)	0.27 (0.31)	4.83 (7.53)	6.63 (3.12)
**CD3+**	mean (SD)	0.03 (0.06)	2.72 (6.76)	0.61 (0.81)	6.99 (8.51)	19.92 (15.79)

Mean and standard deviations of cell numbers per spleen, femur or µl of blood
