# Supplementary material for: Development of LT-HSC-Reconstituted Non-Irradiated NBSGW Mice for the Study of Human Hematopoiesis In Vivo
Source: Front Immunol. 2021 Mar 25;12:642198. doi: 10.3389/fimmu.2021.642198 (PMC8044770; doi:10.3389/fimmu.2021.642198)
Supplement: Supplementary file 10 [file Table_4.pdf]

Supplementary tables

Supplementary table 4. Absolute numbers of T cell subsets in spleens of NBSGW mice 20-22 weeks after humanization with $250x10^3$ $hCD133^+$ UCB HSPCs.

	Tn	Tcm	TemRA	Tem
Absolute number of splenic hCD4+ cells (Mean±SD)	24506±12497	68667± 54468	21609±26484	122877±123907
Absolute number of splenic hCD8+ cells (Mean±SD)	12307±3777	15413±13128	24404±23640	109980±118099

The absolute numbers of $hCD4^+$ or $hCD8^+$ cells in spleens of NBSGW mice 20-22 weeks after humanization with $250x10^3$ $hCD133^+$ UCB HSPCs were 237658+215304 and 162103±156083. Values shown are the means ± S.D.
